# Outcomes of patients with inflammatory breast cancer by hormone receptor- and HER2-defined molecular subtypes: A population-based study from the SEER program

**DOI:** 10.18632/oncotarget.17217

**Published:** 2017-04-19

**Authors:** Juanjuan Li, Yue Xia, Qi Wu, Shan Zhu, Chuang Chen, Wen Yang, Wen Wei, Shengrong Sun

**Affiliations:** ^1^ Department of Breast and Thyroid Surgery, Renmin Hospital of Wuhan University, Wuhan, Hubei, P. R. China; ^2^ Department of Urology, Renmin Hospital of Wuhan University, Wuhan, Hubei, P. R. China

**Keywords:** outcomes, inflammatory breast cancer, molecular subtypes, SEER program

## Abstract

**Background:**

The aim of this study was to evaluate the outcomes of patients with inflammatory breast cancer (IBC), with emphasis on the role of molecular subtypes and radiotherapy.

**Methods:**

A retrospective cohort study to investigate overall survival (OS) and breast cancer-specific mortality (BCSM) in patients with IBC was conducted using data obtained by the Surveillance, Epidemiology, and End Results (SEER) program from 2010–2013. Cox multivariate regression was used to calculate the adjusted Hazard Ratios (aHR).

**Results:**

403 patients were eligible for this study. Patients in the group with hormone receptors (HR)+/HER2- subtype had an OS of 79.6% compared with 89.0 % in the group with (HR)+/HER2+ subtype and 76.8% in the HR-/HER2+ group and 62.9% in the triple-negative (TN) group. BCSM was 16.3% for the HR+/HER2- group, 9.8% for the HR+/HER2+ group, 21.7% for the HR-/HER2+ group, and 30.5% for the TN group. For distant metastases, the results showed that there was a high probability of bone metastasis in HR-positive groups, brain and liver metastasis in HER2-positive groups, and lung metastasis in the TN group. Multivariate analysis demonstrated that estrogen receptor and HER2 positivity were associated with better survival and that the TN subtype had a poorer OS and BCSM compared with other subtypes (P<0.05). Furthermore, patients who received radiotherapy were more likely to have improved survival (P< 0.05).

**Conclusion:**

Inflammatory breast cancer appears to alter the prognosis in association with the receptor status and molecular subtypes. Radiotherapy was still considered to be a crucial treatment for patients with IBC.

## INTRODUCTION

Inflammatory breast cancer (IBC) is an uncommon and extremely aggressive subtype of breast cancer, representing approximately 1% to 5% of all breast malignancies [1]. IBC is characterized by the involvement of the skin and is associated with a poor prognosis [2]. In spite of multimodal therapy, currently, IBC still has much lower median survival times and local recurrence rates as high as 50% compared with other common breast cancers [2, 3]. Moreover, IBC may be less familiar to clinicians and may be misdiagnosed as a dermatologic condition, resulting in a delay in diagnosis.

Because IBC is rare, the biological characteristics of IBC have seldom been reported and the molecular alterations resulting in poor prognosis are not well understood. However, some studies suggest that IBC possesses distinct clinicopathological and molecular features [4]. However, hormone receptors (HR) and human epidermal growth factor receptor-2 (HER2), which define the molecular status of IBC according to immunohistochemistry (IHC), are the fundamental markers that are used to demonstrate molecular features, predict the prognosis and optimize therapeutic regimens. By analyzing the molecular components, the majority of IBCs can be categorized as HR+/HER2-, HR+/HER+, HR-/HER2+, and triple-negative (TN) by IHC. It has been demonstrated that the four molecular subtypes reveal the prognostic discrepancy in both common breast cancers and IBS [2, 5, 6]. However, no population-based studies have investigated the associations between the molecular subtype and survival outcomes in IBC.

Therefore, the current large multi-institutional study using the Surveillance, Epidemiology, and End Results (SEER) aims to evaluate the impact of the receptor status and molecular subtypes on the survival outcomes of patients with IBC.

## RESULTS

### Clinical and tumor characteristics

A total of 403 breast cancer patients were eligible during the 2010–2013 study period. We excluded patients whose survival times were classified as unknown from the analysis. A total of 147 patients in the HR+/HER2- group, 82 in the HR+/HER+ group, 69 in the HR-/HER2+ group and 105 in the TN group had information available and were included in this study.

Differences in patient demographics, cancer characteristics, treatments and outcomes within subgroups are summarized in Table 1. Compared with the HR+/HER2- group, the subgroup of age under 65 years accounted for 82.9% and 81.2% of patients in the HR+/HER+ and HR-/HER2+ groups, respectively. Further, there were more black patients within the TN group than in the other groups. In addition, patients in the HR-/HER2+ and TN groups were universally poorer when compared with HR+/HER2- group (each p<0.05). In terms of metastases, the results showed that the incidence of bone metastasis in HR-positive groups was up to 23.4 % and was significantly higher than that in the HR-negative groups. Additionally, brain and liver metastasis was more frequently observed in the in the HER2-positive groups compared with the HER2-negative groups. Additionally, TN had a significantly high probability of lung metastasis.

**Table 1:** Patient characteristics within subgroups

Variables	HR+/HER2- N=147(%)	HR+/HER2+ N=82(%)	HR-/HER2+ N= 69(%)	TN N=105(%)	P value*
**Follow-up(months)**					
**Age at diagnosis, y**					0.179
**< 35**	6(4.1)	6(7.3)	5(7.2)	4(3.8)	
**35-49**	30(20.4)	19(23.2)	15(21.7)	29(27.6)	
**50-64**	62(42.2)	43(52.4)	36(52.2)	44(41.9)	
**≥65**	49(33.3)	14(17.1)	13(18.8)	28(26.7)	
**Race**					0.488
**white**	121(82.3)	71(86.6)	57(82.6)	78(74.3)	
**Black**	17(11.6)	6(7.3)	7(10.1)	16(15.2)	
**Other**	9(6.1)	5(6.1)	5(7.2)	10(10.5)	
**CHSDA Region**					0.474
**East**	57(38.8)	37(45.1)	23(33.3)	39(37.1)	
**Northern Plains**	6(4.1)	3(3.7)	3(4.3)	9(8.6)	
**Pacific Coast**	76(51.7)	35(42.7)	39(56.5)	51(48.6)	
**Southwest**	6(4.1)	7(8.5)	4(5.8)	6(5.7)	
**Alaska**	2(1.4)	0(0)	0(0)	0(0)	
**Grade**					**0.003**
**Well**	6(4.1)	0(0)	1(1.4)	0(0)	
**Moderately**	48(32.7)	24(29.3)	10(14.5)	15(14.3)	
**Poorly**	65(44.2)	43(52.4)	42(60.9)	60(57.1)	
**Undifferentiated**	24(16.3)	13(15.9)	15(21.7)	27(25.7)	
**Unknown**	4(2.7)	2(2.4)	1(1.4)	3(2.9)	
**Laterality**					0.233
**Left**	76(51.7)	44(53.7)	38(55.1)	57(54.3)	
**Right**	67(45.6)	38(46.3)	31(44.9)	48(45.7)	
**Bilateral**	4(2.7)	0(0)	0(0)	0(0)	
**Stage**					0.742
**III**	118(80.3)	62(75.6)	54(78.3)	86(81.9)	
**IV**	29(19.7)	20(24.4)	15(21.7)	19(18.1)	
**Node stage**					0.934
**N0**	26(17.7)	14(17.1)	17(24.6)	21(20.0)	
**N1**	55(37.4)	35(42.7)	28(40.6)	40(38.1)	
**N2**	31(21.1)	16(19.5)	13(18.8)	16(15.2)	
**N3**	32(21.8)	16(19.5)	10(14.5)	26(24.8)	
**NX**	3(2.0)	1(1.2)	1(1.4)	2(1.9)	
**Distant metastasis**					0.742
**M0**	118(80.3)	62(75.6)	5478.3)	86(81.9)	
**M1**	29(19.7)	20(24.4)	15(21.7)	19(18.1)	
**Bone**	15(10.2)	11(13.4)	6(8.7)	7(6.7)	
**Brain**	0(0)	3(3.7)	1(1.4)	3(2.9)	
**Lung**	5(3.4)	5(6.1)	3(4.3)	7(6.7)	
**Liver**	4(2.7)	8(9.8)	4(5.8)	5(4.8)	
**Tumor size(mm)**					0.884
**≤ 10**	6(4.1)	2(2.4)	4(5.8)	3(2.9)	
**10-20**	8(5.4)	8(9.8)	3(4.3)	8(7.6)	
**20-50**	39(26.5)	20(24.4)	12(17.4)	23(21.9)	
**> 50**	50(34.0)	25(30.5)	25(36.2)	37(35.2)	
**Unknown**	44(29.9)	27(32.9)	25(36.2)	34(32.4)	
**Radiotherapy**					0.662
**No**	48(32.7)	25(30.5)	27(39.1)	35(33.3)	
**Yes**	99(67.3)	59(72.0)	42(60.9)	70(66.7)	
**Treatment**					0.690
**Mastectomy**	142(96.6)	76(92.7)	65(94.2)	97(92.4)	
**BCS+R**	5(3.4)	6(7.4)	4(5.7)	8(7.6)	
**Status**					**< 0.001**
**Alive**	117(79.6)	73(89.0)	53(76.8)	66(62.9)	
**Dead**	30(20.4)	9(11.0)	16(23.2)	39(37.1)	
**Breast cancer**	24(16.3)	8(9.8)	15(21.7)	32(30.5)	
**Other**	6(4.1)	1(1.2)	1(1.4)	7(6.7)	

* P values calculated by Pearson Chi squared testing; Bold if statistically significant, P< 0.05

*y: years, mm: millimeter, y: years, BCS: breast-conserving surgery, HR: hormone receptor, TN: triple negative*.

### Survival analysis

A weighted Kaplan–Meier analysis was used to determine overall survival (OS) and breast cancer-specific mortality (BCSM) in the groups based on molecular subtype, HER2 status and history of radiotherapy. At follow-up, patients in the HR+/HER2- group had an OS of 79.6% compared with 89.0 % in the group with the HR+/HER2+ subtype, 76.8% in the HR-/HER2+ group and 62.9% in the TN group. Moreover, BCSM was 16.3% for the HR+/HER2- group, 9.8% for the HR+/HER2+ group, 21.7% for the HR-/HER2+ group, and 30.5% for the TN group.

**Figure 1:**
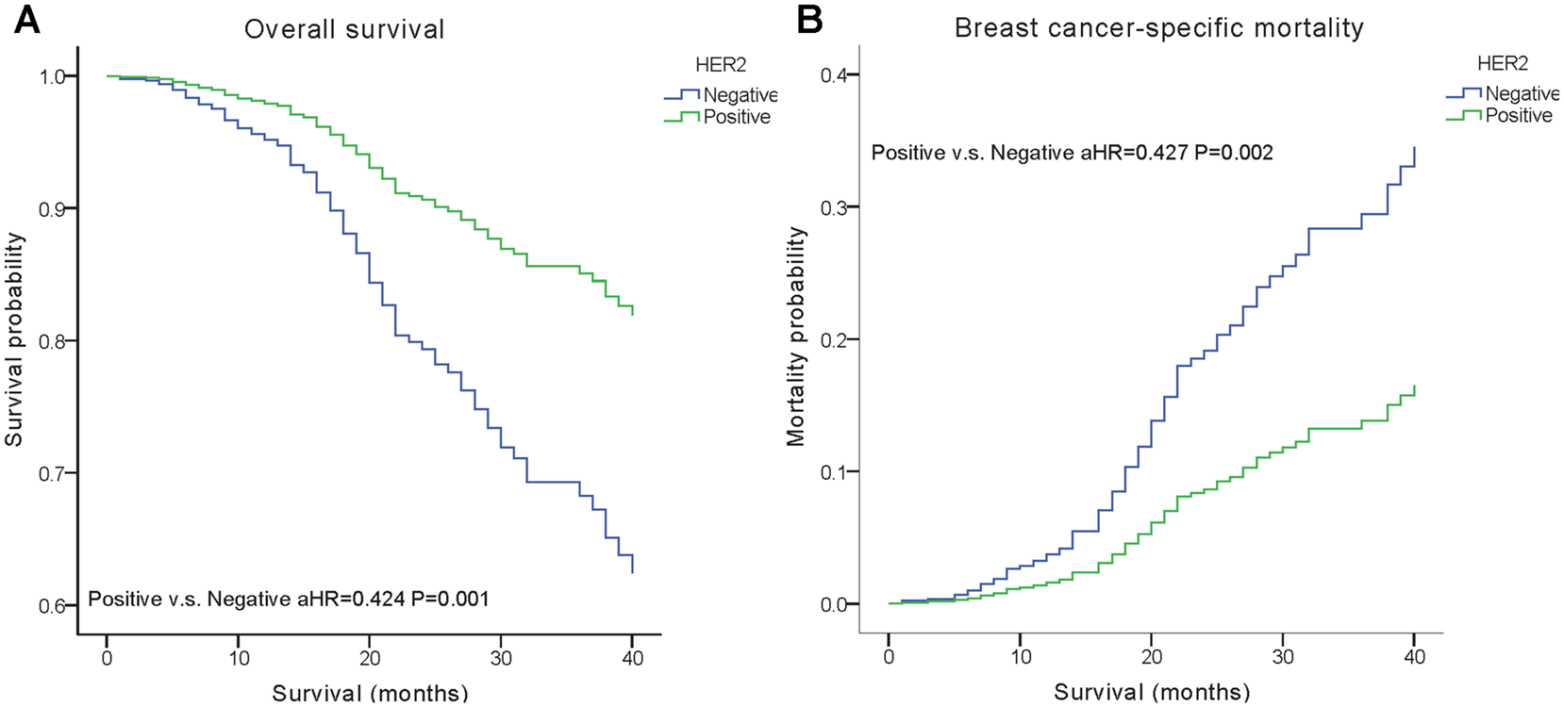
Weighted Kaplan–Meier curves of overall survival (OS) and breast cancer-specific mortality (BCSM) based on the HER2 status **(A)** OS is based on the HER2 status. **(B)** BCSM is based on the HER2 status.

**Figure 2:**
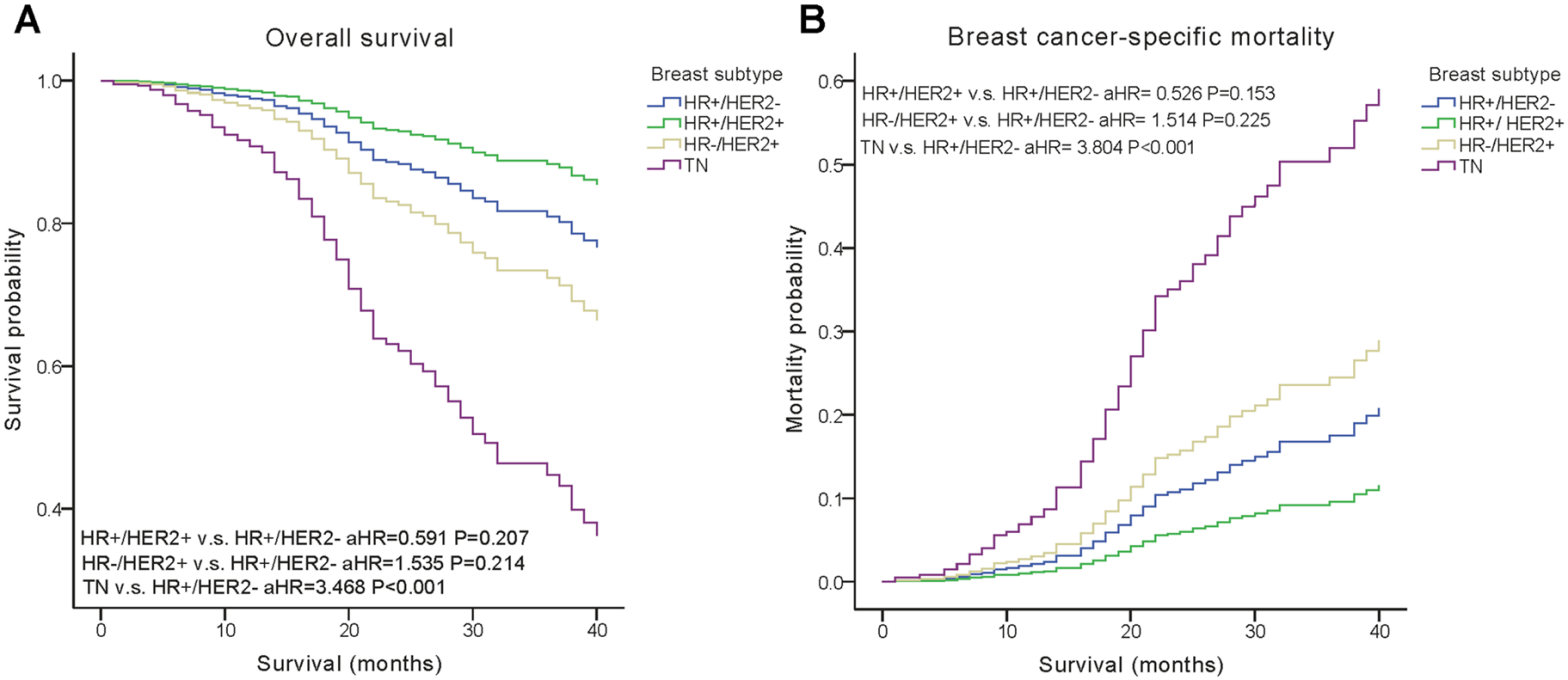
Weighted Kaplan–Meier curves of overall survival (OS) and breast cancer-specific mortality (BCSM) based on the molecular subtypes **(A)** OS is based on the molecular subtypes. **(B)** BCSM is based on the molecular subtypes.

**Figure 3:**
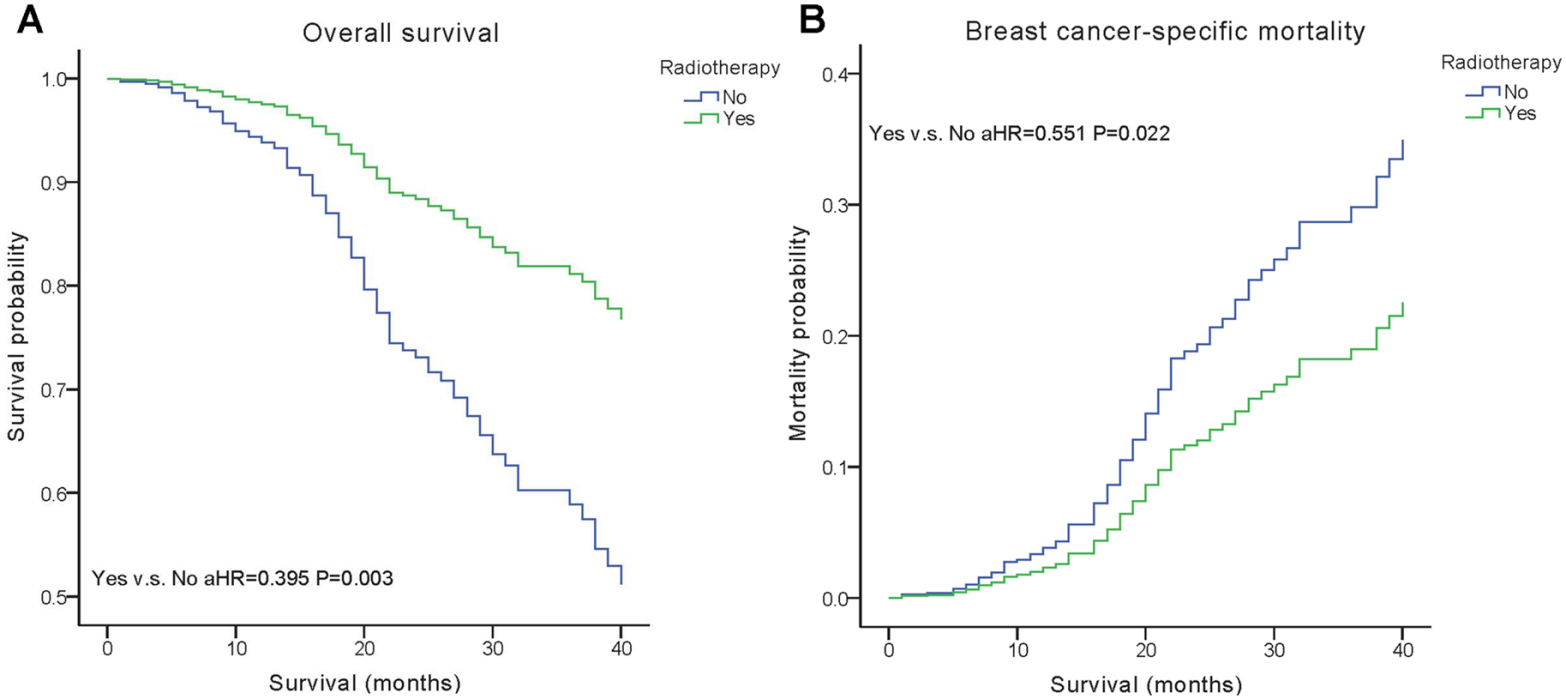
Weighted Kaplan–Meier curves of overall survival (OS) and breast cancer-specific mortality (BCSM) based on radiotherapy **(A)** OS is based on radiotherapy. **(B)** BCSM is based on radiotherapy.

We performed multivariate analysis based on the Kaplan–Meier results. All of the prognostic factors predicted OS and BCSM on multivariate analysis (Table 2). Multivariate analysis was performed using the Cox regression model, demonstrating that HER2 positivity was associated with a better survival (OS, adjusted Hazard Ratios (aHR)=0.424; BCSM, aHR= 0.427, P< 0.05) (Figure 1). The same results were found for the estrogen receptor (ER). Adjusting for other factors, the TN subtype had a poorer OS (aHR=3.468, P<0.001) and BCSM (aHR=3.804, P<0.001) compared with other subtypes (Figure 2). Eventually, we adopted analysis for the options of radiotherapy, showing that patients who received radiotherapy were more likely to have an improved survival and a decreased mortality (P< 0.05) (Figure 3).

**Table 2:** Cox proportional hazards regression model analysis of overall survival (OS) and breast cancer-specific mortality (BCSM)

Variables	OS		BCSM	
	aHR (95% CI)	P-value	aHR (95% CI)	P-value
**Age at diagnosis, y**				
**< 35**	Reference		Reference	
**35-49**	1.827(0.528,6.321)	0.341	1.709(0.487,5.992)	0.406
**50-64**	1.171(0.349,3.921)	0.798	1.016(0.296,3.483)	0.951
**≥65**	2.089(0.617,7.077)	0.237	1.797(0.519,6.229)	0.369
**Race**				
**white**	Reference		Reference	
**Black**	1.583(0.870,2.880)	0.927	1.824(0.944,3.526)	0.074
**Grade**				
**Well**	Reference		Reference	
**Moderately**	1.204(0.657,2.208)	0.318	1.043(0.537,2.026)	0.902
**Poorly**	1.421(0.664,3.041)	0.479	1.336(0.583,3.060)	0.493
**Undifferentiated**	2.899(0.826,10.175)	0.097	3.812(0.950,15.299)	0.059
**Laterality**				
**Left**	Reference		Reference	
**Right**	0.609(0.382,0.971)	**0.037**	0.677(0.411,1.114)	0.125
**Stage**				
**III**	Reference		Reference	
**IV**	3.905(2.414,6.316)	**P<0.001**	5.222(3.062,8.905)	**P<0.001**
**Tumor size(mm)**				
**≤ 10**	Reference		Reference	
**10-20**	0.599(0.139,2.582)	0.492	0.333(0.059,1.880)	0.213
**20-50**	0.773(0.242,2.468)	0.663	0.513(0.152,1.728)	0.282
**> 50**	1.097(0.360,3.336)	0.871	0.761(0.242,2.394)	0.641
**Node stage**				
**N0**	Reference		Reference	
**N1**	1.757(0.873,3.538)	0.114	1.896(0.849,4.233)	0.213
**N2**	1.584(0.245,2.388)	0.223	2.920(0.744,11.454)	0.282
**N3**	1.457(0.386,5.65)	0.939	2.278(1.096,4.733)	0.027
**ER**				
**Negative**	Reference		Reference	
**Positive**	0.300(0.163,0.549)	**P<0.001**	0.272(0.141,0.527)	**P<0.001**
**PR**				
**Negative**	Reference		Reference	
**Positive**	1.015(0.528,1.951)	0.965	0.982(0.487,1.981)	0.960
**HER2**				
**Negative**	Reference		Reference	
**Positive**	0.424(0.253,0.711)	**0.001**	0.427(0.246,0.741)	**0.002**
**Subtype**				
**HR+/HER2-**	Reference		Reference	
**HR+/HER2+**	0.591(0.261,1.338)	0.207	0.526(0.218,1.269)	0.153
**HR-/HER2+**	1.535(0.781,3.016)	0.214	1.514(0.741,3.090)	0.225
**TN**	3.468(2.016,5.965)	**P<0.001**	3.804(2.056,7.039)	**P<0.001**
**Radiotherapy**				
**No**	Reference		Reference	
**Yes**	0.395(0.243,0.640)	**0.003**	0.551(0.331,0.919)	**0.022**
**Treatment**				
**Mastectomy**	Reference		Reference	
**BCS+R**	0.898(0.181,4.462)	0.898	2.458(0.838,7.205)	0.101

* P values calculated by Log-rank testing; Bold if statistically significant, P< 0.05

*BCS: breast-conserving surgery, R: Radiotherapy, HR: hormone receptor, TN: triple negative*.

*aHR: adjusted hazard ratio (adjusted for age at diagnosis, race, grade, histology, tumor size, laterality, ER, PR, HER2, subtype, radiotherapy and treatment)*.

## DISCUSSION

In this large population-based cohort of women diagnosed with IBC, we found a better survival in the ER- and HER2-positive groups compared with those with negative receptors. In our series, cases with the TN subtype had a poorer survival than other subtypes. In addition, our analysis of the adjuvant radiotherapy of IBC demonstrated that radiotherapy could improve prognosis and reduce the mortality for all patients.

In the current study, patients with ER positivity tended to have a greater chance of improved survival compared to those with ER negativity. In the study by Zhou et al. [7], 67 cases of IBC without distant metastases were analyzed and the 2-year OS was evaluated. The results showed that patients with HR-positive IBC had a significantly better survival (median OS 31 months) compared to those with HR-negative IBC (20 months). Further, they attributed the poor survival of patients to the TN subtype. However, the study group had a similar overall survival rate to matched controls for HER2 status. However, we analyzed commonly recognized prognostic factors for patient matching, including HER2 status. We demonstrated that patients with HER2 positivity had a better OS with those with HER2 negativity, adjusting for other prognostic factors. This result was different from Zhou's study and might be due to different variables used for matching in the two studies. In addition, the small sample size in Zhou's study may have caused the difference in results, and the TN subtype included in HER2-negative group may have resulted in survival discrepancy. As expected, we found that patients with the TN subtype had the poorest survival of all subtypes. These findings were consistent with the observations in our study [7–9]. An early study revealed that the 5-year OS rate was 42.7% for IBC patients with the TN subtype, and the loco-regional relapse and distant relapse rate were 38.6% and 56.7%, respectively. All factors were dramatically higher than in the other subtypes [8]. Thus, it was demonstrated that IBC was also a heterogeneous disease with variant molecular subtypes associated with distinct prognostic outcomes as other common breast cancers, and systemic therapies such as endocrinotherapy and anti-HER2 targeted therapy were effective for the treatment of IBC.

For distant metastasis, there was a high probability of bone metastasis in the HR-positive groups, brain and liver metastasis in the HER2-positive groups, and lung metastasis in the TN group. Consistent with these observations, the findings revealed that the incidence of bone recurrence in patients with IBC was up to 28%, while the incidence of recurrence in the central nervous system (CNS), lung, and liver was 21%. Furthermore, the high recurrence rate of the central nervous system was found in the HER2-positive and TN subtypes [10]. The results implied that there was a special association between distant metastasis or recurrence and the molecular subtype. This suggested that individualized strategies were needed for earlier detection or prevention of metastases to improve long-term prognosis.

The therapeutic approach to IBC was multimodal, including primary systemic chemotherapy followed by mastectomy and radiation therapy. Simultaneously, the potential utility of other adjuvant therapies was determined by receptor status, such as those that target HER2 and/or HR [11]. Radiotherapy (RT) represented a particularly effective non-surgical local treatment to achieve the favorable local control. Several studies revealed that preoperative and/or postoperative radiotherapy was an appropriate treatment for IBC patients [12–16]. Greenwalt et al. [16] reported the long-term follow-up results of radiotherapy in patients with IBC. The 15-year local control rate for breast recurrence as a component of first failure was 78%, the regional control rate was 92%, and the local-regional control rate was 74%. The 10-year survival rate for the preoperative RT group was 25% compared to 35% in the postoperative RT group. These studies suggested that radiotherapy was a feasible alternative for IBC patients. The surgical treatment of IBC patients has been controversial. Historically, patients with IBC were predominately treated with mastectomy. With neoadjuvant chemotherapy or RT, some patients achieved complete pathological response (pCR), and breast-conserving surgery (BCS) was applied to the treated IBC patients. One study found that IBC patients who had a good response to systemic therapy may treated with BCS [17]. However, the number of cases receiving BCS plus RT in our study was small. Therefore, a larger series of observations and studies should be conducted to further confirm these results.

A better understanding of the molecular biology of IBC will result in developmental therapeutic approaches and improve survival. According to the molecular profiles of IBC, some studies confirmed that tumors from IBC activated NF-κB to accumulate pro-inflammatory cytokines [18], lost Wnt-inducible signaling protein 3 to activate insulin-like growth factor signaling [19], upregulated the Rho C GTPase gene [20] and so on. Simultaneously, a high level of expression of epithelial growth factor receptor (EGFR) in IBC was associated with a poor prognosis [21], and lapatinib followed by surgical resection or chemotherapy could increase the clinical response rate in HER2+ IBC patients [22]. In addition, the proliferation of endothelial cells was much higher as was the vascular density in IBC patients compared to non-IBC patients, which suggested that antiangiogenic therapies may have potentially greater sensitivity [23]. Nahleh et al. reported that bevacizumab, the monoclonal antibody against vascular endothelial growth factor (VEGF)-A, was used as a neoadjuvant treatment for IBC. The results showed that the addition of bevacizumab combined with dose-dense chemotherapy significantly improved the pCR rate and event-free survival in IBC patients with TN [24]. Therefore, more molecular studies are required to promote significant advancements in treatment.

The major limitations of this study were its retrospective design and small and heterogeneous population. Our survival analysis was limited by a lack of information on systemic therapy and the limited follow-up period. In addition, the sites of distant metastases, including bone, brain, lung and liver, are recorded in the SEER database after 2010, but other metastatic sites were not recorded in detail. Despite these limitations, our study demonstrates that IBC appears to alter prognosis associated with the receptor status and molecular subtypes. Meanwhile, radiotherapy was still considered to be a crucial treatment for patients with IBC.

## PATIENTS AND METHODS

### Data source and study design

We obtained data from the National Cancer Institute's Surveillance, Epidemiology, and End Results (SEER) program between 2010 and 2013. SEER started collecting HER2 status in 2010. Therefore, we used that year as the starting point for our study. We extracted all cases with IBC diagnosed within SEER using the International Classification of Diseases for Oncology, 3rd edition (ICD-O-3) histopathology codes corresponding to inflammatory carcinoma (code 8530). We selected cases with known hormone receptor (HR) status and HER2 status. The following were excluded: individuals diagnosed at autopsy or on a death certificate and patients who did not receive surgery or whose type of surgery was unknown.

Women were categorized as receiving BCS (surgery of primary site variable values of 20–24) and mastectomy (surgery of primary site variable values of 30–80). The demographic variables included age at diagnosis (<35, 35–49, 50–64, ≥ 65 years) and race (white, black, other). The cancer characteristics included stage (III, IV, unknown), grade (well differentiated, moderately differentiated, poorly differentiated, undifferentiated, unknown), N stage (N0, N1, N2, N3, NX, NA), distant metastasis (M0, M1, NA), laterality (right, left, paired, bilateral, unknown), and HR and HER2 status (positive, negative unknown). The treatment characteristics included receipt of radiotherapy (no, yes, unknown). Tumor subtypes were classified as HR+/HER2-, HR+/HER+, HR-/HER2+, and triple-negative (TN) subtypes according to the breast subtype variable.

The two main outcomes in our study were OS and BCSM. Vitality status was recorded as “alive” or “dead” in the SEER dataset. Survival time (in months) was calculated for each patient using the “Completed Months of Follow-Up” option in the SEER database. OS was determined by comparing males and females who were alive at the end of the study period or who were alive at their last follow-up. BCSM was determined by comparing males and females whose cause of death was due to BC with males and females who were alive at the end of the study period, had died due to other causes, or who were alive at their last follow-up. Cases without survival times were classified as unknown and removed from the study.

### Statistical analysis

Patient demographics and cancer- and treatment-related characteristics were compared between females and males using the Chi square or Fisher's exact tests as appropriate. Within each variable, patients with unknown data were excluded from the comparative analysis. A matched subgroup analysis was performed. Survival probabilities for OS and BCSM were estimated using the weighted Kaplan–Meier method, and variables were compared using the log-rank test in the subgroups. Multivariate Cox proportional hazard regressions were used to obtain HRs and their respective 95% confidence intervals and show the strength of the estimated relative risk; these approaches were applied to model the relationship between potential covariates and either OS or BCSM. All statistical analyses and all charts of survival probabilities were performed using SPSS 19.0 (IBM Corporation, Armonk, NY, USA). A two-sided P value < 0.05 was considered statistically significant.
